# Two Distinct Lesions of Inverted Follicular Keratosis of the Scalp: A Case Report

**DOI:** 10.7759/cureus.31055

**Published:** 2022-11-03

**Authors:** Didik S Heriyanto, Vincent Lau, Verdy Phang

**Affiliations:** 1 Department of Anatomical Pathology, Faculty of Medicine, Public Health, and Nursing, Universitas Gadjah Mada/Dr. Sardjito General Hospital, Yogyakarta, IDN

**Keywords:** elderly patients, scalp lesions, inverted follicular keratosis, immunohistochemistry, human papillomavirus (hpv)

## Abstract

Inverted follicular keratosis (IFK) is a rare benign tumor characterized by endophytic growth on the follicular infundibulum. IFK clinically and pathologically resembles other malignant lesions such as squamous and basal cell carcinomas due to similar basosquamous proliferation. Hence, the differentiation of these lesions is important as treatments vary substantially. We present the case of a 60-year-old female with two distinct skin lesions on her scalp. The lesions were excised, and the specimens were sent for histopathologic, immunohistochemistry, and human papillomavirus (HPV) testing. Due to the lesion's location, morphological ambiguity, and predilection for the elderly, differentiation from malignancy or viral-infected skin lesions is difficult. Adequate assessment must be done histopathologically to confirm the benign nature of the lesion as IFK presents usually as a singular lesion. Our case report investigates further whether HPV contributes to the development of IFK in this particular case. In this instance, HPV had no contribution to the development of the lesion.

## Introduction

Inverted follicular keratosis (IFK) is a rare benign tumor characterized primarily by endophytic growth on the follicular infundibulum. There have been a few cases of IFK reported in the literature [1-4], However, two distinct IFK lesions on the scalp have never been documented. While IFK can affect any part of the skin, the most common presentation is a solitary verrucous papule in the predilection area of the upper lid or chin. It primarily affects older men. Some reports have demonstrated that IFK is a benign asymptomatic skin lesion and may be an HPV-related irritated seborrheic keratosis that grows inward [1,2].

Clinically and pathologically, IFK can resemble malignant lesions such as squamous and basal cell carcinoma because of basosquamous proliferation. It is crucial to correctly differentiate between IFK and malignancy as their respective treatments vary substantially. Clinically, it also resembles verruca and seborrheic keratosis. Pathologically, the endophytic proliferation of the follicular infundibulum with squamous eddies has been described as an essential finding for IFK diagnosis. However, these histopathologic findings can also be observed in warts, seborrheic keratosis, and basal or squamous cell carcinoma. Hence, further investigation with immunohistochemistry is needed to confirm the lesion's diagnosis and nature. Recent studies and guidelines demonstrate that surgery and imiquimod are highly effective treatments. However, as of now, no specific literature has been published describing two distinct IFK lesions on the scalp [3].

## Case presentation

**Figure 1:**
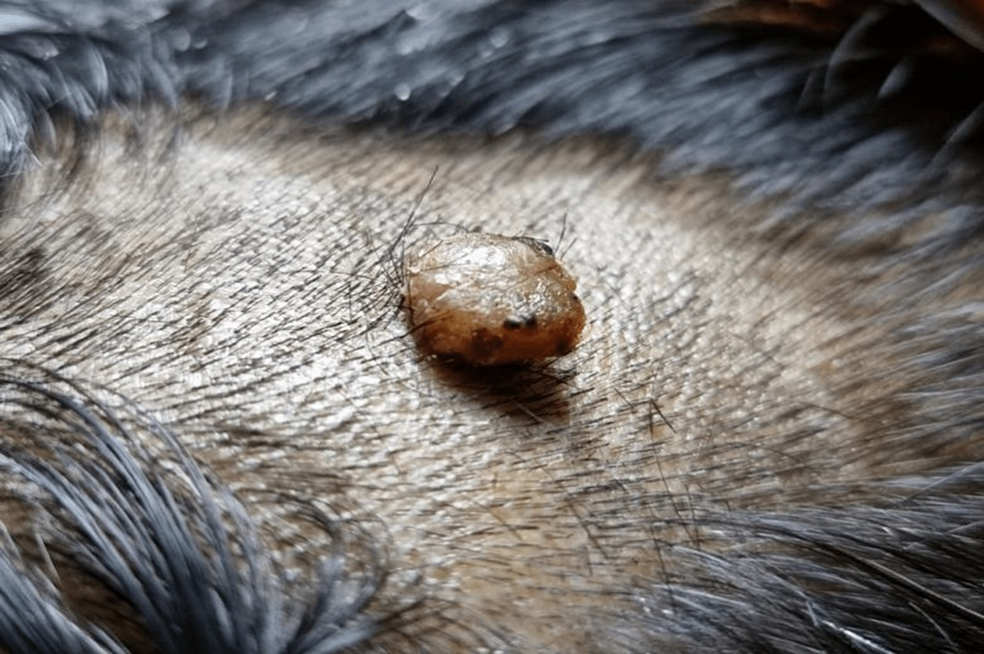
The first lesion, a skin-colored pedunculated mass (1 cm) with a smooth-regular surface and minimal crusting

**Figure 2:**
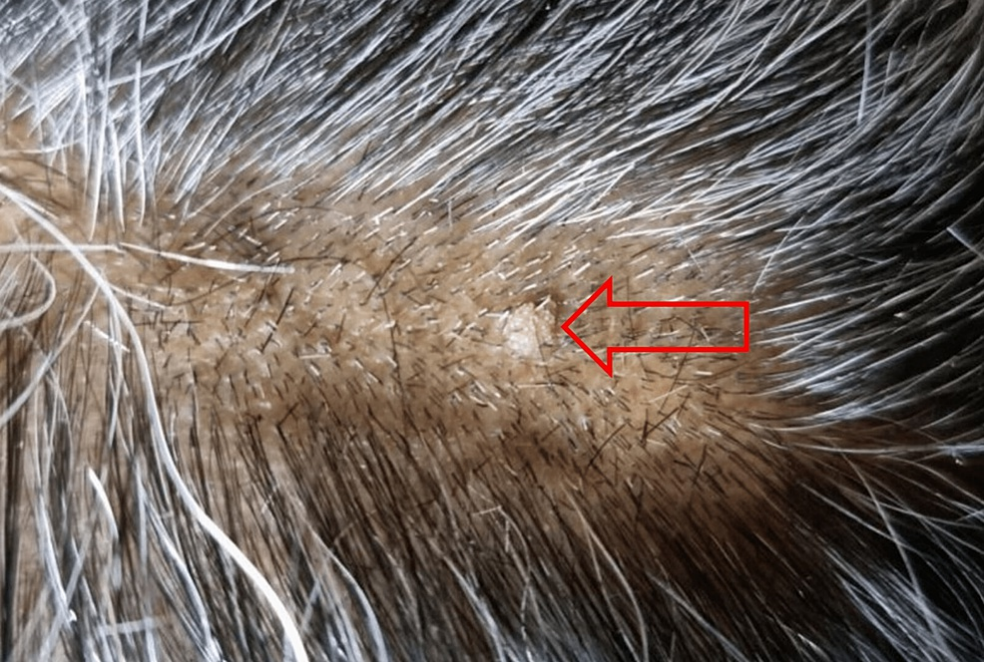
The second lesion, a skin-colored papule (0.3 cm × 0.4 cm) with a verrucous surface (red arrow)

We present a case of a 60-year-old housewife with no significant comorbidities. She was presented to our clinic with two skin lesions on her scalp. The first lesion was located on the vertex. Physical examination revealed a skin-colored pedunculated mass (1 cm) with a smooth-regular surface and minimal crusting (Figure 1). The second lesion was located on the parietal area and clinically appeared as a skin-colored papule (0.3 cm x 0.4 cm) with a verrucous surface (Figure 2). She had noticed the first lesion two years ago and the second lesion six months ago. The lesions were not growing rapidly. The lesions were excised with a simple elliptical technique under local anesthesia. The specimens were sent for histopathologic examination and human papillomavirus (HPV) testing.

**Figure 3:**
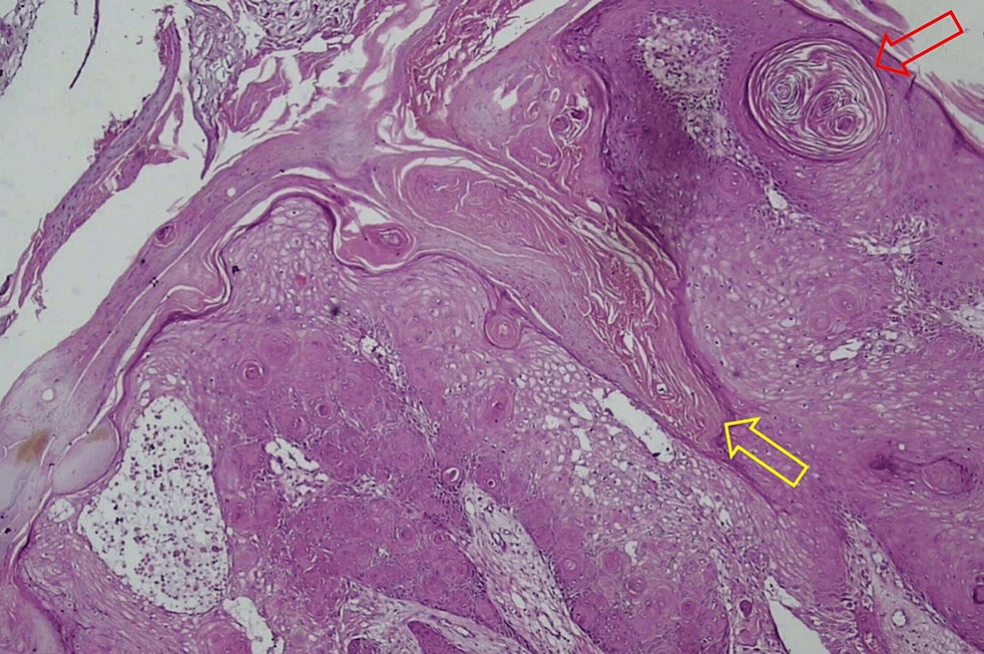
Photomicrograph of the first lesion showed skin tissue with squamous cell proliferation that forms distinct nests with endophytic growth toward the dermis (yellow arrow) and horn cysts (red arrow) (H&E, 40X) H&E: Hematoxylin and eosin.

**Figure 4:**
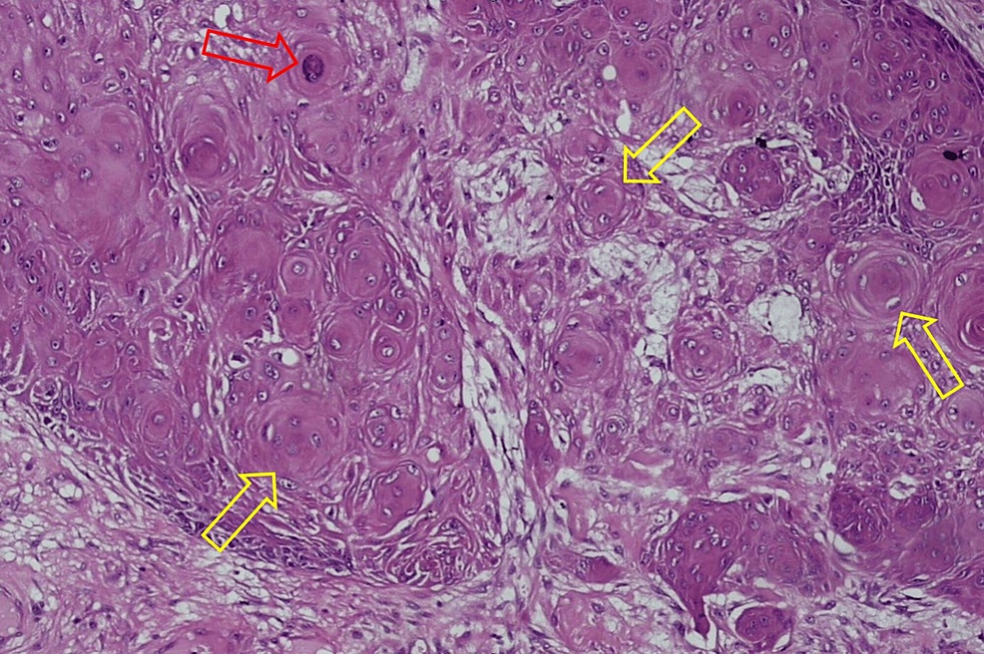
Photomicrograph of the first lesion showing concentric circles of keratin (red arrow) with squamous eddies (yellow arrows) (H&E, 100X) H&E: Hematoxylin and eosin.

**Figure 5:**
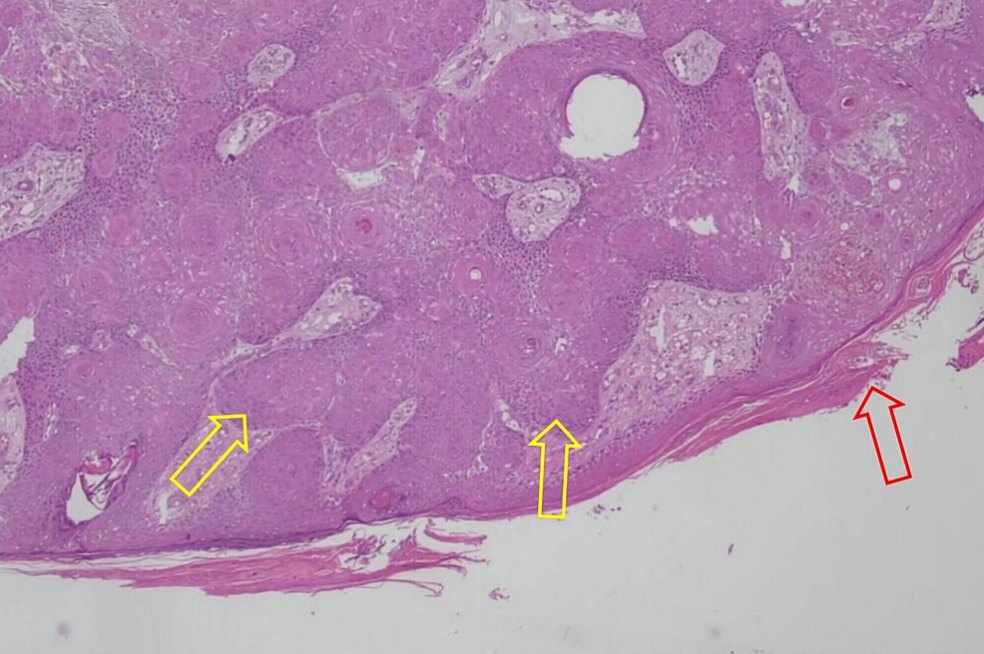
Photomicrograph of the second lesion showing skin tissue with stratified bulbous squamous cell proliferation (yellow arrows) and hyperkeratotic foci (red arrow) (H&E, 40X) H&E: Hematoxylin and eosin.

**Figure 6:**
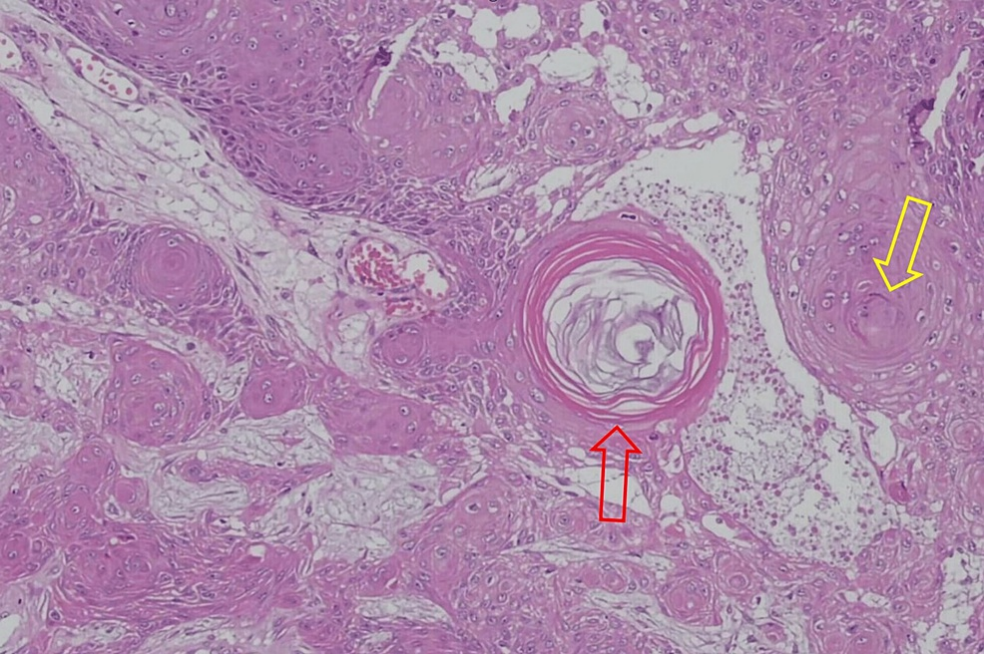
Photomicrograph of the second lesion showing horn cysts (red arrow) and concentric circles of keratin with squamous eddies (yellow arrow) (H&E, 100X) H&E: Hematoxylin and eosin.

**Figure 7:**
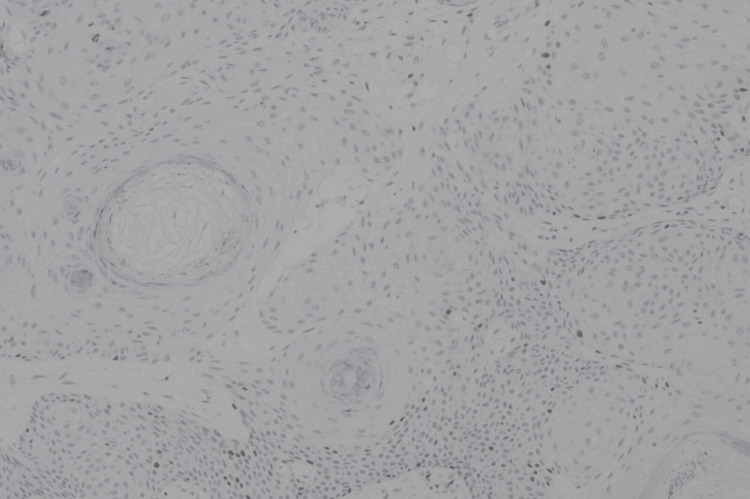
Immunohistochemistry with anti-Ki67 antibody showed low expression of Ki-67 proliferation index for the first lesion (anti-Ki-67 antibody IHC, 40X) Ki-67: Kiel-67; IHC: Immunohistochemistry.

**Figure 8:**
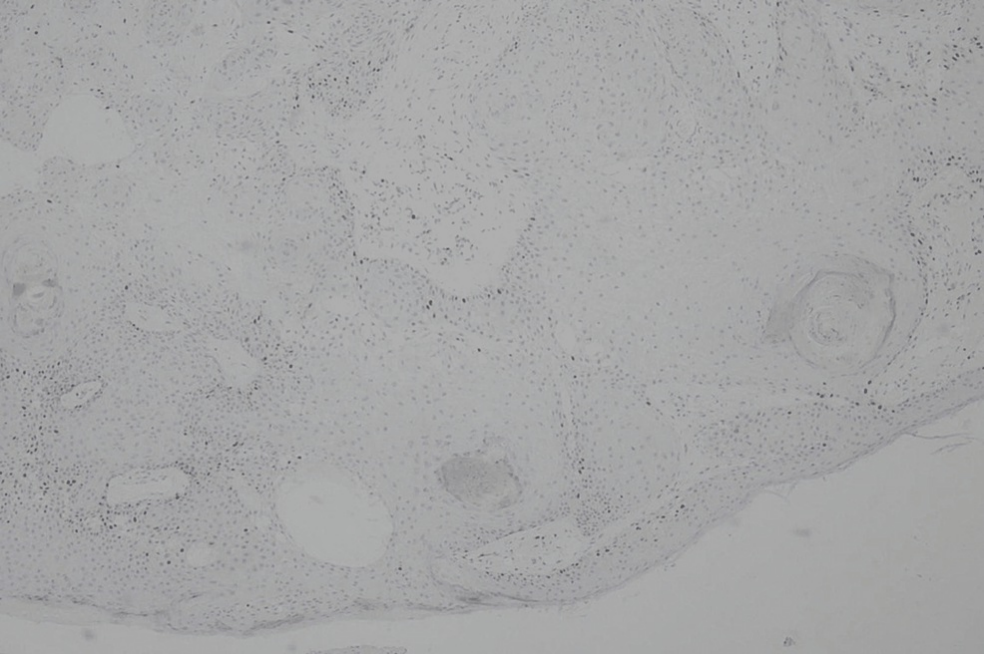
Immunohistochemistry with anti-Ki67 antibody showed low expression of Ki-67 proliferation index for the second lesion (anti-Ki-67 antibody IHC, 40X) IHC: Immunohistochemistry; Ki-67: Kiel-67.

The results of the histopathological examination for the first lesion showed skin tissue with squamous cell proliferation that forms distinct nests with endophytic growth toward the dermis and horn cysts (Figure 3). Concentric circles of keratin with squamous eddies were observed in the first lesion (Figure 4). The histopathological examination for the second lesion showed skin tissue with stratified bulbous squamous cell proliferation and hyperkeratotic foci (Figure 5). Horn cysts and concentric circles of keratin with squamous eddies can also be observed (Figure 6). Dermal stroma with moderate infiltration of lymphocytes and plasma cells with no signs of malignancy were found on both lesions. Immunohistochemistry (IHC) examination with anti-Ki67 antibody for the first lesion showed low expression of Ki-67 proliferation index (Figure 7), while IHC with anti-Ki67 antibody for the second lesion also showed low expression of Ki-67 proliferation index (Figure 8). Deoxyribonucleic acid (DNA) extraction was performed on formalin-fixed, paraffin-embedded (FFPE) tissue preparations. The DNA samples were assayed using the HPV genotyping detection kit, and a quantitative polymerase chain reaction (qPCR) was performed. The test showed negative results for HPV DNA. During a 10-month evaluation, the patient showed no recurrence after complete treatment.

## Discussion

IFK is an uncommon benign tumor characterized by endophytic growth, predominantly on the follicular infundibulum [4]. The most common presentation is a single, non-pigmented verrucous papule on the face, typically appearing in middle-aged or elderly individuals and predominantly in men. Approximately, 90% of cases involve the head and neck [5]. The most likely differential diagnoses for a lesion on the scalp such as in our case are warts, seborrheic keratosis, or nevus. Seborrheic keratoses and actinic keratoses are becoming increasingly common with age, and the latter develops especially as hair thinning occurs. Clinicians should also be aware of other possible pathologies as differential diagnoses, such as malignant lesions in the elderly. These malignant lesions include basal and squamous cell carcinomas. Even though only 1%-2% of scalp tumors are malignant, they account for approximately 13% of malignant cutaneous tumors. Literature suggests that a lesion on the scalp might have a higher chance to be a malignancy, especially in older age and those who are often exposed to the sun [6]. This raises a concern with our finding of two distinct lesions on the scalp, especially in an elderly patient.

Because of the nature of the lesion, we decided to excise the lesion with a simple elliptical technique under local anesthesia. The diagnosis of IFK is uncommon and is generally established histopathologically as clinical differentiation from other lesions is difficult [7]. Extra caution should be noted as common clinical and pathological differentials includes warts, keratoacanthoma, squamous cell carcinoma, and basal cell carcinoma [4]. The specimens were sent for histopathologic examination, and for both lesions, the findings are consistent with the diagnosis of IFK. In other literature [4], IFK of the upper eyelids has been associated with plugs of keratin, a dense inflammatory response characterized by inverted papillomatosis, a number of squamous eddies, and acanthosis. In this instance, there were no cellular atypia, nests of cells, or pathologic mitotic observed [4]. It was also described that the histopathology examination for IFK may reveal stratified, squamous epithelium foci with hyperkeratosis and parakeratosis. Another report histopathologically describes that IFK exhibits an endophytic, somewhat bulbous proliferation of eosinophilic keratinocytes with basaloid or squamous differentiation. Squamous eddies and horn cysts are also commonly seen [7].

Our case is unique because we observed two clinically distinct lesions of IFK in unusual locations, yet these lesions had similar histopathological findings. As the diagnosis of a lesion could harbor numerous clinical findings, histopathology, immunohistochemistry, and molecular pathology examinations should aid in the diagnosis of these lesions. According to some cases, the upper eyelid is a common predilection area for IFK [4,8]. A study reported a single, well-defined, asymptomatic, skin-colored plaque along the upper eyelid margin. The surface was irregular, verrucous, and crusted and lacked pliability. The growth of the plaque has been gradual over the past three months [8]. In contrast to our case, no other lesions were present elsewhere, while our case has two distinct lesions on the scalp. Our case should give a new insight into the predilection and natural history of the disease IFK since eyelids and scalp have different skin characteristics and different degrees of exposure to irritation or even viral infections.

It is reported that HPV might contribute to the development of IFK [8]. Some literature has also disapproved of the claims [9,10]. The lesions have a similar presentation to a wart with an unusual location. We decided to perform the HPV DNA examination to further explore the nature and confirm the diagnosis of the lesion. If both lesions were positively assessed for HPV, it could be an important finding for future research about the association between HPV and IFK. According to different studies [11], IHC has a sensitivity of 52%-87% for the detection of HPV. In that particular study, they recommend performing a more sensitive test such as PCR in order to strongly reject the role of HPV in IFK lesions [11]. We performed the DNA examination with qPCR, and our findings suggest that these lesions are not caused by HPV. We also performed an IHC examination for anti-Ki-67 antibody to confirm the benign nature of the lesion, in which our findings confirmed that both lesions have a low proliferation index. The findings importantly ruled out basal and squamous cell carcinoma as the differential diagnosis.

Complete surgical excision is the typical method for treating IFK. No invasive growth or metastasis has been reported in the literature following surgical excision [2]. The finding is in accordance with our case on the 10-month follow-up for the patient, which showed no recurrence. In the literature, the patient was advised to apply imiquimod cream [4]. We did not have imiquimod as a choice of treatment because it has not yet been registered in the Indonesian national formulary, and its availability is really limited. Dermoscopy is not available in our setting. In the literature, dermoscopy may show yellow-white structureless amorphous areas with central keratinous plugs, white lines, red dots (blood spots), milky red areas, and hairpin vessels surrounded by a whitish halo [4].

**Figure 9:**
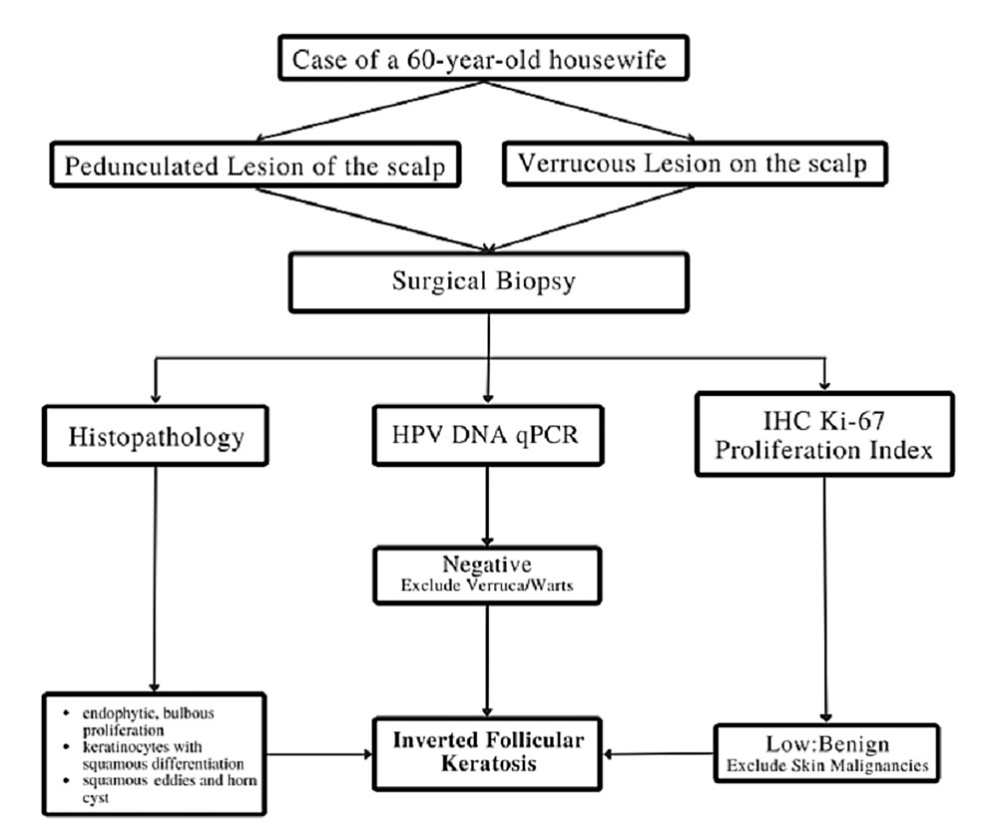
Stepwise approach on the basis of examinations performed on this particular case

Lastly, owing to its site, morphological ambiguity, and predilection for the elderly, IFK may be mistaken as a malignant growth or viral-infected skin lesion. This is a cause for concern in the patient, and adequate assessment regarding the benign nature of the lesion must be done with histopathological evidence. Additionally, examinations such as HPV DNA detection and proliferation index represented by ki-67 should be performed whenever possible. Even though rare, IFK should be considered as a differential among conditions like squamous cell carcinoma, basal cell carcinoma, seborrheic keratosis, and verruca. On the basis of examinations performed, we devised a stepwise approach that we used to arrive at a definitive diagnosis for this particular case of IFK (Figure 9) [3]. Often, dermatology cases resembling these lesions are more of a cosmetic concern for a patient. As physicians, we must always consider the underlying cause as these lesions may resemble malignant lesions [7].

## Conclusions

We report a case of two distinct lesions of IFK on the scalp that may be mistaken as a malignant growth or viral-infected skin lesion. In the diagnosis of skin lesions, many clinical manifestations may differ from case to case, even within the same individual. Histopathology, immunohistochemistry, and molecular pathology examinations aid in the definitive diagnosis of these lesions. As a physician, we must always consider the underlying causes so that we can treat the patient accordingly.
